# Reductions in hypothalamic *Gfap* expression, glial cells and α-tanycytes in lean and hypermetabolic *Gnasxl*-deficient mice

**DOI:** 10.1186/s13041-016-0219-1

**Published:** 2016-04-14

**Authors:** Andrew P. Holmes, Shi Quan Wong, Michela Pulix, Kirsty Johnson, Niamh S. Horton, Patricia Thomas, João Pedro de Magalhães, Antonius Plagge

**Affiliations:** Integrative Genomics of Ageing Group, Institute of Integrative Biology, University of Liverpool, Crown Str, Liverpool, L69 7ZB UK; Cellular and Molecular Physiology, Institute of Translational Medicine, University of Liverpool, Crown Str, Liverpool, L69 3BX UK

**Keywords:** Gnas, Genomic imprinting, Hypothalamus, Tanycyte, Gfap, Glia, Energy homoeostasis, RNAseq

## Abstract

**Background:**

Neuronal and glial differentiation in the murine hypothalamus is not complete at birth, but continues over the first two weeks postnatally. Nutritional status and Leptin deficiency can influence the maturation of neuronal projections and glial patterns, and hypothalamic gliosis occurs in mouse models of obesity. *Gnasxl* constitutes an alternative transcript of the genomically imprinted *Gnas* locus and encodes a variant of the signalling protein Gα_s_, termed XLα_s_, which is expressed in defined areas of the hypothalamus. *Gnasxl*-deficient mice show postnatal growth retardation and undernutrition, while surviving adults remain lean and hypermetabolic with increased sympathetic nervous system (SNS) activity. Effects of this knock-out on the hypothalamic neural network have not yet been investigated.

**Results:**

RNAseq analysis for gene expression changes in hypothalami of *Gnasxl*-deficient mice indicated *Glial fibrillary acid protein* (*Gfap*) expression to be significantly down-regulated in adult samples. Histological analysis confirmed a reduction in Gfap-positive glial cell numbers specifically in the hypothalamus. This reduction was observed in adult tissue samples, whereas no difference was found in hypothalami of postnatal stages, indicating an adaptation in adult *Gnasxl*-deficient mice to their earlier growth phenotype and hypermetabolism. Especially noticeable was a loss of many Gfap-positive α-tanycytes and their processes, which form part of the ependymal layer that lines the medial and dorsal regions of the 3^rd^ ventricle, while β-tanycytes along the median eminence (ME) and infundibular recesses appeared unaffected. This was accompanied by local reductions in Vimentin and Nestin expression. Hypothalamic RNA levels of glial solute transporters were unchanged, indicating a potential compensatory up-regulation in the remaining astrocytes and tanycytes.

**Conclusion:**

*Gnasxl* deficiency does not directly affect glial development in the hypothalamus, since it is expressed in neurons, and Gfap-positive astrocytes and tanycytes appear normal during early postnatal stages. The loss of Gfap-expressing cells in adult hypothalami appears to be a consequence of the postnatal undernutrition, hypoglycaemia and continued hypermetabolism and leanness of *Gnasxl*-deficient mice, which contrasts with gliosis observed in obese mouse models. Since α-tanycytes also function as adult neural progenitor cells, these findings might indicate further developmental abnormalities in hypothalamic formations of *Gnasxl*-deficient mice, potentially including neuronal composition and projections.

**Electronic supplementary material:**

The online version of this article (doi:10.1186/s13041-016-0219-1) contains supplementary material, which is available to authorized users.

## Background

The hypothalamus exerts important regulatory functions in whole-body energy homeostasis, including food intake, energy expenditure and SNS activity [1–3]. A neuronal network between several hypothalamic nuclei, including the arcuate nucleus (Arc), dorsomedial nucleus (DMH) and paraventricular nucleus (PVH), integrates peripheral hormonal signals like leptin, insulin and ghrelin, which reflect levels of energy reserves and feeding status. The pathways of leptin action, to inhibit food intake and stimulate energy expenditure when adipose tissue fat reserves are high, have been elucidated in some detail. Leptin inhibits orexigenic neuropeptide Y (NPY) neurons and activates anorexigenic pro-opiomelanocortin (POMC) neurons in the arcuate nucleus [3]. This leads to activation of the melanocortin system (neurons expressing MC4 and MC3 receptors) in the PVH, parabrachial nucleus, dorsal vagal complex and the intermediolateral cell column of the spinal cord. While MC4R-expressing neurons in the PVH and parabrachial nucleus mainly regulate food intake, those in other hypothalamic nuclei, the dorsal vagal complex and the spinal cord have been implicated in the control of energy expenditure and SNS activity [1–3].

In rodents, the formation of the hypothalamic neural network is incomplete at birth as, for example, axonal projections from the Arc to the PVH and DMH develop and mature over the first two weeks postnatally [4–6]. Leptin has an important neurotrophic function during this postnatal period. Deficiency of leptin results in diminished POMC and NPY/Agrp projections from the Arc [4]. Full functionality of the neuronal circuits controlling food intake and energy expenditure is established by weaning age. Apart from leptin deficiency, nutritional status at embryonic or postnatal stages can also impact on the formation of neural circuits controlling energy homeostasis [5, 7]. Perturbations during these developmental stages do not always lead to obesity as, for example, postnatal undernutrition results in permanent leanness, transformation of white to brown-like adipose tissue, increased hypothalamic leptin and melanocortin sensitivity and increased leptin receptor expression [8–10]. The changes in molecular mechanisms and neural networks leading to such permanent leanness have not yet been investigated in detail.

Apart from neuronal networks, glial cells and tanycytes, which are located within the ependymal layer of the 3^rd^ ventricle, also participate in the hypothalamic regulation of energy homeostasis and are responsive to metabolic status [11–14]. Both cell types form protrusions that are in contact with blood capillaries, or the ventricular surface in the case of tanycytes, and neurons, thus enabling the transport and exchange of nutrients, neurotransmitters and hormones. A responsiveness of these non-neuronal cells to whole-body energy status is indicated by data showing hypothalamic gliosis and increased *Gfap* expression in genetically or diet-induced obese models [15–20]. Furthermore, recent work has highlighted a role for tanycytes as neural progenitor cells that contribute to the hypothalamic energy homeostasis regulating network [21–24]. Different subsets of tanycytes show a neurogenic response to specific signalling factors as well as to a high-fat diet [24–28].

**Fig. 1:**
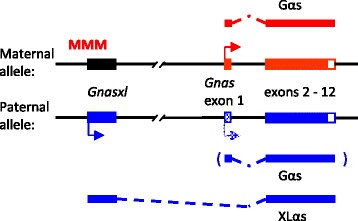
Simplified scheme of the imprinted *Gnas* locus. The *Gnas* and *Gnasxl* transcripts, which encode the Gα_s_ and XLα_s_ proteins, respectively, are shown with their alternative promoters and splicing arrangements. Promoter activities on the two parental alleles are indicated by arrows. *Gnasxl* expression is suppressed by DNA methylation (MMM) on the maternal allele. *Gnas* is biallelelically expressed in most tissues, but silenced on the paternal allele is some cell types (hatched box and arrow). For simplicity, other features of the locus, e.g. non-coding RNAs, are not shown

The *Gnas* locus, which encodes variants of the G-protein α-stimulatory subunit (Gα_s_), is involved in the hypothalamic regulation of energy balance [29]. The locus consist of a complex arrangement of protein-coding and non-coding transcripts, which are expressed from alternative promoters (Fig. 1) [30, 31]. In addition to the well-known Gα_s_ protein, an NH_2_-terminal variant (extra-large α_s_; XLα_s_) is expressed from a separate promoter and transcript (*Gnasxl*). Both proteins stimulate cAMP production after activation of seven-transmembrane receptors, but XLα_s_ can also exert a Gα_q/11_-like function and increase inositol 1,4,5-trisphosphate signalling in some cell types [32, 33]. The *Gnas* locus is epigenetically regulated by a mechanism termed genomic imprinting, which results in monoallelic gene expression according to parental origin [30]. DNA methylation acquired in the female germline silences the expression of *Gnasxl* on the maternally inherited allele. By contrast, *Gnas* remains biallelically expressed in most tissues of the offspring, apart from a few cell types, including specific brain regions, in which the paternally inherited *Gnas* allele is silenced (Fig. 1) [29–31].

Due to the monoallelic expression of these transcripts, heterozygous knock-out (KO) mutations were used to analyse the roles of *Gnas* and *Gnasxl* in energy homeostasis, which revealed opposite phenotypes [29, 34]. Brain-specific knock-out of *Gnas* expression from the maternal allele results in obesity due to disrupted melanocortin receptor/Gα_s_ signalling and consequently reduced energy expenditure [35, 36]. By contrast, deletion of *Gnasxl* expression (from the paternal allele, *Gnasxl*^m+/p-^) causes a lean and hypermetabolic phenotype associated with increased energy expenditure and elevated SNS activity [37–39]. This phenotype of adult KO mice follows an impaired postnatal development, which is characterised by growth retardation, reduced feeding, hypoglycaemia and lack of adipose reserves [38]. XLα_s_ is expressed in defined regions of the brain at neonatal and adult stages; its neonatal expression in some peripheral tissues is, however, down-regulated towards weaning [32, 38–40]. Within the hypothalamus, *Gnasxl* is present in neurons of the Arc, DMH, PVH, lateral hypothalamus and the suprachiasmatic nucleus (SCN) [40]. It is currently unclear how *Gnasxl* functions within the neural energy homeostasis network, which receptor(s) might signal via XLα_s_ in the brain, and whether the postnatal development of the hypothalamic network might be impaired as a consequence of the neonatal undernutrition experienced by *Gnasxl*^m+/p-^ pups.

Whole transcriptome sequencing (i.e., RNAseq) has been shown to have many advantages for gene expression profiling when compared to microarrays [41], including in the context of the rodent brain [42]. Taking an unbiased approach to analyse gene expression changes in hypothalami of *Gnasxl*-deficient mice via RNAseq, we identified a 2-fold down-regulation of the glial marker *Gfap*. This unexpected finding was confirmed by histological analyses of adult hypothalami, which showed reduced Gfap-positive cell numbers, while initial glial development at neonatal stages was not different. The loss of glial cells in adult hypothalami appears to be a consequence associated with the lean *Gnasxl*-deficient phenotype, and our observations complement opposite findings of gliosis in obese mouse models. Furthermore, we show not only astrocytes, but also Gfap-expressing α-tanycytes to be diminished. Since α-tanycytes have a role as neural progenitor cells, our data point towards potentially more far-reaching impacts on the development or maintenance of the hypothalamic energy homeostasis network.

## Results

### Transcriptome analysis indicates reduced *Gfap* expression in hypothalami of *Gnasxl*-deficient mice

To take an unbiased approach to screen for changes in gene expression in hypothalami of lean and hypermetabolic *Gnasxl*^m+/p-^ mice, we undertook a RNAseq transcriptome analysis using the Illumina platform. Hypothalamus RNA from 6 *Gnasxl*^m+/p-^ mice and 6 WT littermates was isolated and two samples of identical genotype were combined to obtain a total of 3 *Gnasxl*^m+/p-^ and 3 WT RNA sample pools for library preparations and sequencing. The six resulting sequencing files were mapped individually using TopHat 2.0.4 to the mm10 build of the mouse genome. Using the Cufflinks package 2.0.2, we identified a total of 332 significantly differentially expressed genes (Additional file 1; Gene Expression Omnibus (GEO) accession number GSE75878). Most of these changes were within the range from 4-fold increase to 4-fold decrease, although some small nucleolar RNAs showed particularly high fold-changes (Additional file 1). Notably *Gnasxl* RNA, which is lost in the KO mice [38], was not identified as differentially expressed. This is most likely due to the complex structure of the *Gnas* locus with its transcript-specific alternative promoters and mostly shared exons [30, 31], which are difficult to resolve. To analyse the dataset of differentially expressed genes further for association with common processes and pathways [41], a functional enrichment analysis was undertaken and revealed statistically significant enriched categories amongst overexpressed genes related to two broad categories: ribosomes, rRNA binding and translation; and mitochondria and electron transport chain (Additional file 2). No statistically significant categories were found amongst down-regulated genes after correcting for multiple hypotheses testing (Additional file 2).

**Fig. 2:**
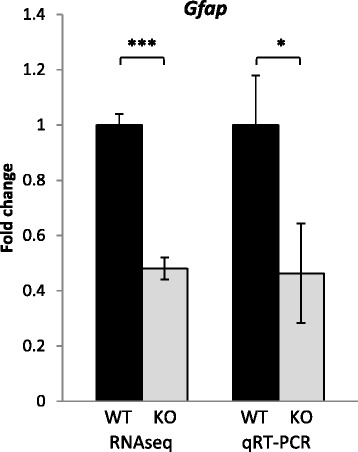
Reduced *Gfap* RNA expression levels in *Gnasxl* ^m+/p-^ hypothalami. A similar 2-fold down-regulation of *Gfap* RNA levels was found by RNAseq and qRT-PCR in *Gnasxl* knock-out hypothalami (***p < 0.0001; *p < 0.05; Student’s independent *t*-test; *n* = 3 WT and 3 KO pools of RNA with each pool consisting of two individual samples of the same genotype)

From the RNAseq list of genes (Additional file 1), we selected a subset, which have been implicated in the literature with hypothalamic regulation of energy homeostasis, for further verification by qRT-PCR. The candidate gene *Glial fibrillary acidic protein* (*Gfap*) showed a similar 2-fold down-regulation by RNAseq and qRT-PCR (*Gnasxl*^m+/p-^ RNAseq fold change: 0.48 ± 0.04 SEM, *p* < 0.0001, *n* = 3 per genotype; qRT-PCR fold change: 0.46 ± 0.18 SEM; *p* < 0.05, *n* = 3 per genotype) (Fig. 2). Since this down-regulation of hypothalamic *Gfap* RNA in lean and hypermetabolic *Gnasxl*^m+/p-^ mice contrasts with the up-regulation and gliosis found in obese mouse models [15–20], we decided to further investigate Gfap protein expression on the histological level.

### Reduced glial cell numbers in adult, but not postnatal, hypothalami of *Gnasxl*^m+/p-^ mice

**Fig. 3:**
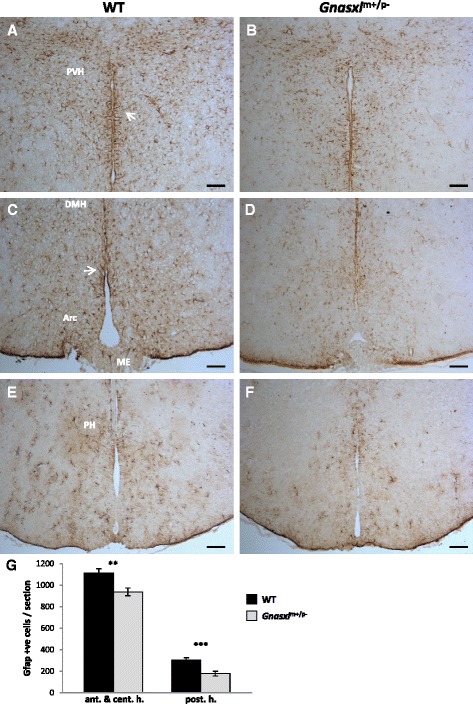
Lower Gfap-positive cell counts in hypothalami of adult *Gnasxl* ^m+/p-^ mice. IHC for Gfap on coronal brain sections from WT (**a**, **c**, **e**) and KO (**b**, **d**, **f**) littermates at different rostro-caudal levels. In addition to astrocytes, the α-subset of ependymal tanycytes and their processes (white arrows) were found to express Gfap. **a** and **b** Anterior-dorsal hypothalamus at the level of the Paraventricular Nucleus (PVH). **c** and **d** Central-ventral hypothalamus at the level of the Arcuate Nucleus (Arc). **e** and **f** Level of the posterior hypothalamus (PH). Scale bar = 100 μm. **g** Quantification of Gfap-expressing cells at the anterior-central and posterior levels, respectively, (anterior & central: WT = 1110 ± 42 vs KO = 937 ± 35 cells/section ± sem, **p < 0.01, *t*-test, *n* = 11 matched pairs of sections from 5 WT and 5 KO mice; posterior: WT = 305 ± 21 vs KO = 179 ± 23 cells/section ± sem, ***p < 0.001, *t*-test, *n* = 11 matched pairs of sections from 5 WT and 5 KO mice)

**Fig. 4:**
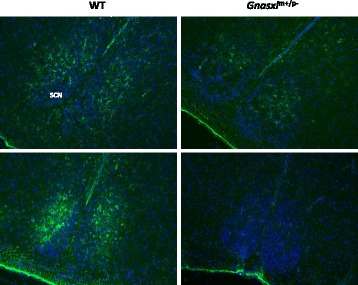
Fewer Gfap-expressing astrocytes in the suprachiasmatic nucleus (SCN). Gfap-immunofluorescence (green) in the SCN of two adult WT and *Gnasxl* ^m+/p-^ littermates (nuclear stain: DAPI). Images taken at original magnification 100 x

**Fig. 5:**
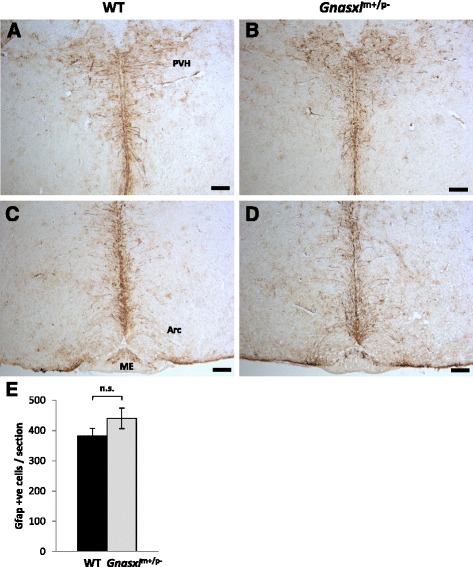
Glial cell numbers are not different in hypothalami of postnatal *Gnasxl* ^m+/p-^ mice. Gfap IHC on postnatal day 15 brain sections from WT (**a** and **c**) and KO (**b** and **d**) littermates. **a** and **b** Anterior-dorsal hypothalamus at the level of the PVH; (**c** and **d**) central-ventral hypothalamus at the level of the Arc. Scale bar = 100 μm. **e** Quantification of Gfap-positive cells: WT = 382 ± 25 vs KO = 440 ± 34 cells/section ± sem, *p* > 0.05, *t*-test, *n* = 33 matched pairs of sections along the rostro-caudal axis of the hypothalami of 3 WT and 3 KO mice

**Fig. 6:**
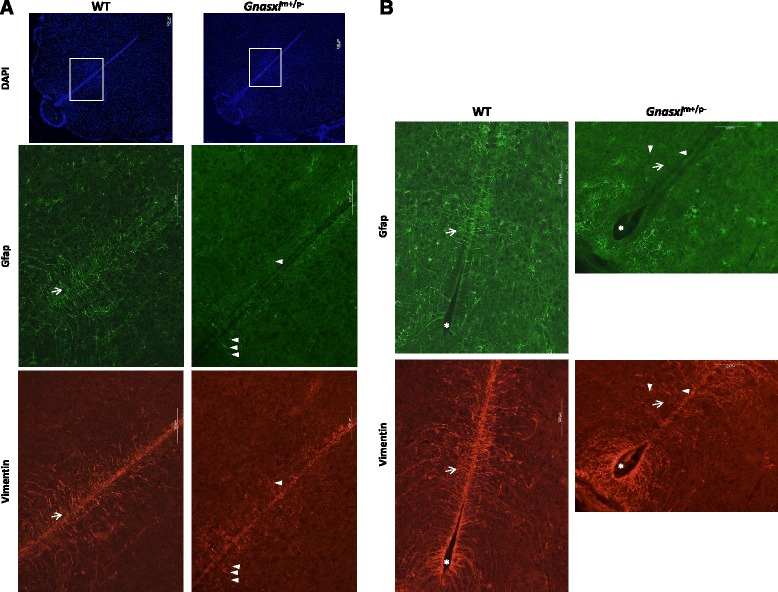
Loss of α-tanycytes. Co-immunofluorescence for the tanycyte marker Vimentin and for Gfap on (**a**) central and (**b**) posterior hypothalamic sections of WT and *Gnasxl*-deficient mice. White arrows mark the general localisation of α-tanycytes, most of which co-express both markers. Asterisks indicate the regions of Gfap-negative, Vimentin-positive β-tanycytes. While double-positive α-tanycyte extensions and cell bodies are strongly reduced, β-tanycytes appear unaffected in the KO mice. White arrowheads indicate remaining Gfap/Vimentin double-positive α-tanycyte processes in the KO samples. The white rectangle on the DAPI images in (**a**) indicates the position of the higher magnification Gfap/Vimentin images. Scale bars represent 100 μm

To assess the localisation and number of hypothalamic Gfap-positive cells, immunohistochemistry (IHC) was carried out on brain sections from adult *Gnasxl* WT and KO littermates. Apart from astrocytes, Gfap was found to be expressed in a subset of periventricular tanycytes and their processes, which extend from the surface of the 3^rd^ ventricle into the hypothalamic parenchyma (Fig. 3, Fig. 5 and Fig. 6) [14]. Only the α1 and α2 subpopulations, which are located along the dorsal ventricle and the Arc, were found to express Gfap [14, 27, 28]. The β1 and β2 tanycytes, which line the ventral ventricle along the infundibular recesses and ME, respectively, were negative for the glial marker. In *Gnasxl*^m+/p-^ samples, we found fewer Gfap-positive cells in all areas along the rostro-caudal and dorso-ventral axes of the hypothalamus, including the PVH, DMH, Arc, ME and PH (Fig. 3a–f). Quantification indicated a 41 % reduction in Gfap-positive cell numbers in posterior areas of the hypothalamus (WT = 305 ± 21 vs KO = 179 ± 23 cells/section ± sem, ***p < 0.001, *t*-test, *n* = 11 matched pairs of sections from 5 WT and 5 KO mice), while anterior and central regions showed 26 % fewer glial cells (WT = 1110 ± 42 vs KO = 937 ± 35 cells/section ± sem, **p < 0.01, *t*-test, *n* = 11 matched pairs of sections from 5 WT and 5 KO mice) (Fig. 3g). In the anterior hypothalamus, the suprachiasmatic nucleus (SCN) also contained fewer Gfap-positive astrocytes (Fig. 4). To investigate whether a reduced number of Gfap-expressing cells is specific to the hypothalamus or occurs generally throughout the brain, we additionally assessed cell numbers in the hippocampus. We found no significant difference in this brain region (WT = 769 ± 16 vs KO = 746 ± 19 cells/section ± sem, *p* > 0.05, *t*-test, *n* = 15 matched pairs of sections from 5 WT and 5 KO mice) (Additional file 3). These findings indicate that a reduction in *Gfap* expression and glial cell numbers is a characteristic feature of the hypothalamus area of *Gnasxl*^m+/p-^ brains.

Since *Gnasxl* is expressed in hypothalamic neurons, but not in glia or the ependymal layer of the hypothalamus (Additional file 4) [40], the reduction in Gfap-expressing cells in the adult KO samples appears to be a consequence of *Gnasxl* deficiency and an adaptation to the severe postnatal undernutrition phenotype of *Gnasxl*^m+/p-^ pups [38, 39]. As glial cell differentiation follows neuronal differentiation and mainly occurs at late embryonic and early postnatal stages [14, 43], we investigated whether this reduction in hypothalamic Gfap-positive cells has a postnatal onset. Histological comparison of Gfap expression in hypothalami of postnatal day 15 (P15) samples did not, however, show a significant difference in cell numbers between the two genotypes (Fig. 5) (WT = 382 ± 25 vs KO = 440 ± 34 cells/section ± sem, n.s. *p* > 0.05, *t*-test, *n* = 33 matched pairs of sections from 3 WT and 3 KO). Earlier postnatal stages (P1, P5 and P10) showed overall lower numbers of Gfap-positive cells, but no difference was found between WT and KO samples (data not shown). These findings indicate that deficiency of *Gnasxl* does not impact on the initial formation and differentiation of glial cells, but that their reduction in adult hypothalami is a consequence of the KO phenotype, which includes postnatal growth retardation and undernutrition (hypoglycaemia, hypolipidemia) as well as increased metabolic rate driven by elevated SNS activity during adult stages [37–39].

### Loss of α-tanycytes and their processes

**Fig. 7:**
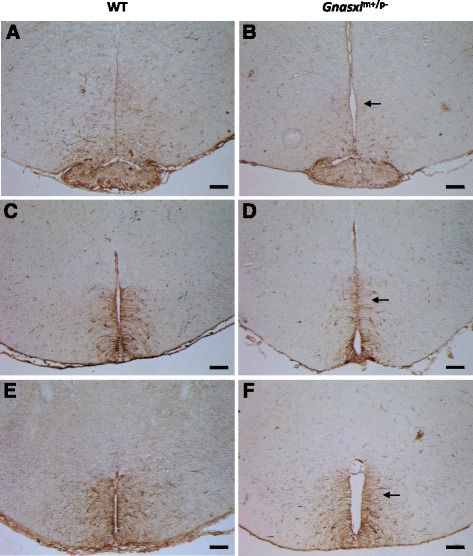
Reduced expression of Nestin in α-tanycyte region. Immunohistochemistry for the neural progenitor cell marker Nestin shows reduced expression in the region of α-tanycytes (arrows), but no difference in the more ventrally located β-tanycyte population. Pairs of images from three different WT and *Gnasxl* ^m+/p-^ adult littermates are shown for medial (**a**, **b**) to posterior (**c, d, e**, **f**) positions of the hypothalamus. Scale bars indicate 100 μm

From the Gfap-IHC (Fig. 3) it appears that not only astrocytes are affected, but also the α-subset of tanycytes. α-tanycytes facilitate the exchange of nutrients and signalling molecules through their long processes, which are in contact with the ventricular surface and bridge to neurons and blood vessels far into the hypothalamic parenchyma [14]. Furthermore, recent reports have associated α-tanycytes with adult neural progenitor cell functions [14, 24, 27, 28]. To further investigate the effect of *Gnasxl*-deficiency on this cell type, we undertook IHC for the intermediate filament Vimentin, which is a marker for all tanycytes as well as ependymocytes [27]. We found a strong reduction in Vimentin-positive tanycyte processes specifically in the α-subset of tanycytes, while the β-subpopulation appeared normal (Fig. 6). Lack of Gfap and Vimentin staining largely coincided in the *Gnasxl*^m+/p-^ samples, which points to a loss of many of the double-positive α-tanycyte processes after postnatal day 15 and is in accordance with the reduced number of Gfap-positive cell bodies (Fig. 3). Remaining Vimentin-positive cells without processes located in the ependymal layer of the α-tanycyte region most likely represent ependymocytes [27]. In line with these findings, a trend towards reduced *vimentin* gene expression, although not significant, was observed in the RNAseq analysis of whole hypothalamus lysates (Additional file 1). Additional investigations into the expression pattern of the neural progenitor cell marker Nestin showed a localised reduction of the protein in the region of α-tanycytes (Fig. 7), similar to the changes observed for Vimentin. Whether these changes in the α-tanycyte population impact on neural progenitor cell functions [14, 24, 27, 28] and hypothalamic network maintenance in *Gnasxl*^m+/p-^ mice remains to be determined.

Astrocytes and tanycytes express specific solute transporter proteins for glucose (*Slc2a1*/*Glut1*) and excitatory amino acids (*Slc1a2*/*Glt1* and *Slc1a3*/*Glast1*), thus regulating their availability and exchange between compartments of the hypothalamic microenvironment [14, 44, 45]. Despite the cellular changes described above we did, however, not detect a reduction in expression levels of these transporter genes in whole hypothalamus lysates of *Gnasxl*^m+/p-^ mice by RNAseq analysis or qRT-PCR (Additional files 1 and 5), which might be due to compensatory up-regulation in the remaining glial cell population.

## Discussion

The unbiased assessment of gene expression changes in hypothalami of adult *Gnasxl*-deficient mice via RNAseq revealed a 2-fold down-regulation of the glial marker *Gfap*, and further histological examination indicated reduced numbers of Gfap-positive astrocytes and α-tanycytes. These findings were unexpected, since *Gnasxl* is not expressed in glial cells or in the ependymal layer of the 3^rd^ ventricle, but is present in neurons of the Arc, DMH, PVH, lateral hypothalamus and the SCN [40]. Furthermore, Gfap-positive cell numbers were not diminished at postnatal stages, when the developmental process of glia and tanycyte differentiation reaches completion in mice [14, 43, 46, 47]. This indicates that the initial formation of these cell types is not impaired by lack of XLα_s_, but that their reduction in adult hypothalami must be a consequence of the phenotype of the *Gnasxl* KO mice, which comprises post-natal under-nutrition, continued hypoglycaemia, reduced body weight and fat mass as well as hypermetabolism [38, 39].

It is remarkable in this context that nutritional programming in rats, i.e. postnatal under-nutrition, results in adult offspring with a very similar phenotype to *Gnasxl*-deficient mice, including reduced body weight and fat mass, transformation of white adipose tissue to metabolically stimulated ‘brown-like’ adipose tissue and hypoleptinemia [8–10, 38, 39]. However, changes in hypothalamic neuronal networks have been little analysed at the histological level in rodent models of postnatal undernutrition [48], and glial or tanycyte abnormalities have not been reported so far. In support of our findings in *Gnasxl*^m+/p-^ mice, some studies on the effects of foetal and/or postnatal protein restriction and malnutrition described similar reductions in Gfap expression and glial cell numbers in the suprachiasmatic nucleus and the medial preoptic area [49, 50]. By contrast, genetic or diet-induced mouse models of obesity have been much better characterised for changes in the hypothalamus. Specifically, observations of gliosis and increased Gfap expression in obese mice contrast with the glial effects we identified in lean *Gnasxl* KOs [15–20]. The gliosis observed in obese mice leads to increased glial ensheathment and reduced numbers of synapses on POMC and NPY neurons in the Arc [18]. Whether, correspondingly, glial processes surrounding hypothalamic neurons in *Gnasxl*-deficient mice are reduced remains to be determined on an ultrastructural level. Any consequences of such glial changes on the activity of the neural network regulating energy homeostasis are only beginning to be explored, although in obese mice a reduced inhibitory synaptic input on POMC neurons was detected [17, 18].

A loss of Gfap expression in *Gnasxl* KO mice was especially evident in the α-subset of tanycytes, which are part of the ependymal layer lining the 3^rd^ ventricle along the Arc, ventromedial hypothalamus and DMH [14]. The β-subset of tanycytes, which is located more ventrally along the infundibular recesses of the 3^rd^ ventricle and the ME, does not express Gfap, but both subsets are marked by expression of Vimentin [13, 14, 27, 28]. While Vimentin IHC of β-tanycytes and their processes appeared undiminished in *Gnasxl*^m+/p-^ mice, it was reduced in a similar pattern to Gfap in the α-tanycyte subset. Few double-positive α-tanycyte processes remained. Some Vimentin-positive/Gfap-negative cells without processes were still detectable in the ependymal layer of the α-tanycyte region of *Gnasxl* KOs, which most likely represent Vimentin-expressing ependymocytes [27]. Although we also detected a trend towards reduced *vimentin* gene expression in the RNAseq experiment, this was not significant in the context of lysates from whole-hypothalamus samples. Overall, these data indicate that a substantial proportion of α-tanycytes have been lost in the hypothalamus of *Gnasxl*-deficient adult mice.

Two major functions of α-tanycytes have been characterised. With their processes these cells bridge the ependymal border of the 3^rd^ ventricle with the Arc, VMH and DMH and are capable of transporting substrates from the cerebrospinal fluid into the hypothalamic parenchyma [11, 13, 14]. It was shown that α-tanycytes express glucose transporters and are sensitive to changes in extracellular glucose concentration, thus implicating them as a functional component in the hypothalamic network that regulates energy homeostasis [11, 13]. We did not detect changes in glial transporter gene expression in our RNA samples from whole hypothalami, which could be due to compensatory up-regulation in the remaining cells. However, at this stage we cannot exclude disturbances in localised nutrient or neurotransmitter transport or availability.

Recent reports have shown that tanycytes also possess neural progenitor cell functions [13, 21, 23]. Both, β- as well as α-tanycytes can proliferate and generate neurons and to some extent glial cells, although β-tanycytes appear to exert this function mainly during juvenile stages and show less proliferation during adulthood [25, 26]. By contrast, α-tanycytes were shown to self-renew and give rise to neurons and glial cells at adult stages, and this process was specifically stimulated by fibroblast growth factors (FGFs) and insulin-like growth factor 1 (IGF-1) [24, 27, 28]. Some α-tanycytes co-express Gfap and the neural stem cell transcription factor Sox2 [28], which showed a trend towards reduced expression levels in our RNAseq analysis of *Gnasxl* KO samples (Additional file 1). For another marker of neural progenitor cells, the intermediate filament protein Nestin, insufficient sequence reads were obtained in the RNAseq analysis, most likely due to low *Nestin* RNA levels, but it was detectable histologically in the ependymal layer. Nestin protein expression appeared to be reduced specifically at the level of α-tanycytes, similar to Vimentin and Gfap. This finding further supports the view that the α-tanycyte-associated neural progenitor cell population has been impacted. However, a functional assessment of hypothalamic stem cells in *Gnasxl* deficient mice and any consequences of a partial loss of α-tanycytes on neural cell maintenance in the hypothalamus remain to be determined. Taking into account the nutritional programming effects discussed above, it can also not be excluded that postnatal undernutrition, either diet-induced [8–10] or as described in our genetic model [38, 39], results in additional changes in the hypothalamic network that regulates energy homeostasis. Future investigations will be aimed at assessing cell proliferation and potential changes in the proportions of specific neuron types in the hypothalamus, for example NPY and POMC neurons in the Arc.

The direct function of *Gnasxl* on a cellular and molecular level in neurons of the brain remains currently unclear. Although the XLα_s_ protein has been shown to couple activated receptors to the cAMP signalling pathway in cell lines [33] and can also mediate G_q/11_-like IP3 signalling in renal proximal tubules [32], the physiological relevant receptors in the brain have not yet been identified. Lack of XLα_s_ leads to severe postnatal undernutrition and failure to grow, which is associated with a high pre-weaning mortality [38]. KO mice that survive into adulthood show increased SNS activity, which causes an elevated basic metabolic rate, body temperature and cardiovascular parameters [37, 39]. Taking into account the data presented here, it appears now likely that the phenotype of *Gnasxl*^m+/p-^ mice that survive into adulthood needs to be considered as a composite of direct effects related to lack of XLα_s_ signalling in neurons, as well as adaptions in the hypothalamic neural network, including astrocytes and α-tanycytes, which occur in response to the severe postnatal undernutrition of the mutant mice. The RNAseq data generated as part of this work will be a useful resource for comparisons with other nutritional programming studies, as well as for further investigations into the functions of *Gnasxl* in the hypothalamus. Additionally, the *Gnasxl* KO mouse model could have relevance to human in both aspects: in the context of rare genetic neonatal growth disorders that are associated with loss of *GNASXL* at chromosome 20q13 [51–55], and as a model to investigate the consequences of neonatal undernutrition on programming of energy homeostasis in human adult life [56].

## Conclusions

Lean and hypermetabolic *Gnasxl*-deficient mice show reduced expression levels of the glial marker *Gfap*, as well as a reduction in astrocyte and α-tanycyte numbers in the hypothalamus at adult stages. The findings that the changes are not present at postnatal stages when these glial cell types are completing their development and, furthermore, that *Gnasxl* is not expressed in these cell types, lead us to conclude that the hypothalamic changes are an adaptation or consequence of the severe neonatal undernutrition the KO mice experience. The adult phenotype of *Gnasxl* KO mice, which includes increased metabolic rate, reduced fat mass, increased glucose tolerance and insulin sensitivity, as well as elevated SNS activity, is reminiscent of rodent models of metabolic programming through postnatal undernutrition. The phenotype of adult *Gnasxl*-deficient mice, therefore, appears to be a combination of lack of XLα_s_ signalling in neurons, as well as adaptations in the hypothalamic network that regulates energy homeostasis and which includes astrocytes and α-tanycytes.

## Methods

### Animal care

The *Gnasxl* mouse line [38] was maintained on a CD1 outbred background. *Gnasxl*-deficient offspring (*Gnasxl*^m+/p−^), which lack XLα_s_ expression from the paternally inherited allele, was produced by mating CD1 females with *Gnasxl* mutation-carrying males. Mice were maintained in the animal facility of the University of Liverpool on a 12 h light – 12 h dark cycle and had unlimited access to water and standard chow diet. All animal work was approved by the Animal Welfare and Ethical Review Body of the University of Liverpool and carried out in accordance with the UK Animals (Scientific Procedures) Act 1986 (UK Home Office Project Licence PPL40/3577) and the directive 2010/63/EU for Europe.

### RNA seq and bioinformatics analyses

Hypothalami were dissected from 6 *Gnasxl*^m+/p-^ and 6 WT littermates (six months old females). Hypothalamic total RNA was isolated using a Qiagen RNeasy Plus Mini kit. The quality of the RNA was confirmed on a Bioanalyzer 2100 (Agilent Technologies) with a RNA Integrity Number of 8.5 or higher for all samples. For cost effectiveness, but to retain biological sample variability, RNA from two *Gnasxl*^m+/p-^ and two WT samples were pooled, respectively, to obtain a total of 3 *Gnasxl*^m+/p-^ and 3 WT RNA pools. Ribosomal RNA (rRNA) was removed from each pool using the Ribominus Eukaryote Kit for RNA‐Seq (Life Technologies), and 95–98 % depletion of rRNA was confirmed on a Bioanalyzer 2100. Before library preparation, the RNA sample concentrations were quantified on a Qubit (Life Technologies) using Ribogreen. For each pooled sample, 50 ng of RNA was used for library preparation using the ScriptSeq v2 RNA‐Seq Library Preparation Kit (Epicentre). Briefly, the RNA was first fragmented at 85 °C for 5 min, random hexamer cDNA synthesis primers with tagging sequences for Illumina sequencing slides were annealed and cDNA prepared using StarScript Reverse Transcriptase. cDNA was purified using the Agencourt AMPure XP system (BeckmanCoulter) and then amplified with ScriptSeq Index PCR Primers (Epicentre), in order to reduce representation bias during multiplex sequencing. Suitable size distribution of the library preparations were confirmed using the Bioanalyzer 2100. Pair-end sequencing (2 x 100 bp reads) of barcoded samples was carried out by the Centre for Genomic Research (University of Liverpool, UK) using an Illumina Genome Analyzer IIx (GAIIx). The data were quality-controlled using a filter for CHASTITY ≥ 0.6 and demultiplexed using the Illumina CASAVA 1.8.2 pipeline. A further quality control analysis was then performed using FastQC (Babraham Bioinformatics). Both sense and anti‐sense FASTQ files were mapped to the mm10 build of the *M. musculus* genome (UCSC genome browser) using TopHat 2.0.4., incorporating a repeatmask (RepeatMasker) [57]. Of the approximately 28 million reads per sample generated, 40 % were mapped to the mouse genome. Data were then analysed using Cufflinks, Cuffcompare and Cuffdiff (version 2.0.2) with multiple hypotheses correction [58] to the GTF transcript annotation file provided by Illumina iGenomes, to examine differential expression between *Gnasxl*^m+/p-^ and WT samples. RNAseq data files have been deposited at Gene Expression Omnibus (GEO) database under accession number GSE75878 (see also Additional file 1). Functional enrichment analysis was performed in the DAVID bioinformatics resources [59] using the whole mouse genome as background and default parameters. Full results, including *p*-values after correcting for multiple hypotheses testing, are available in Additional file 2.

### qRT-PCR

Quantitative RT-PCR was carried out on the same three pooled WT and three pooled *Gnasxl*^m+/p-^ total RNA samples that had been used for the RNAseq experiments. cDNA was synthesised using MMLV Reverse Transcriptase (Life Technologies) and real-time PCR was performed using the Brilliant II SYBR Green QPCR Master Mix with Low Rox (Agilent Technologies) on an Applied Biosystems 7500 Fast Real‐Time PCR System. Relative quantification was calculated through normalisation to two housekeeping genes, β-Actin (*Actb)* and Cyclophilin A (*Ppia*), using the Applied Biosystems 7500 software. Primer efficiencies were evaluated on a five-fold dilution series of cDNA and only primer pairs with 93–107 % efficiency and a R^2^ > 0.98 were used for relative quantification. The following primer pairs were used: *Actb*: For 5’-GCTTCTTTGCAGCTCCTTCGT-3’, Rev 5’-ATATCGTCATCCATGGCGAAC-3’; *Ppia*: For 5’-CAAATGCTGGACCAAACACAA-3’, Rev 5’-GCCATCCAGCCATTCAGTCT-3’; *Gfap*: For 5’-ACCAGCTTACGGCCAACAGT-3’, Rev 5’-CCGAGGTCCTGTGCAAAGTT-3’. Glial solute transporter gene expression was assessed separately on a Biorad CFX Connect instrument using iTaq Universal SYBR Green Supermix reagents (Bio-Rad Laboratories). Hypothalamic RNA from six adult WT and six *Gnasxl*^*m+/p-*^ littermates was used. Target gene data were normalised to two housekeeping genes, β-Actin (*Actb*) and Beta-2-microglobulin (*B2m*), and relative expression was calculated via the ΔCt method. The following primer pairs were used: *Actb*: For 5’-GGCTCCTAGCACCATGAAGATC-3’, Rev 5’-ACATCTGCTGGAAGGTGGACA-3’; *B2m*: For 5’-ATTCACCCCCACTGAGACTG-3’, Rev 5’-GTCTCGATCCCAGTAGACGG-3’; *Slc2a1*: For 5’-CTCACCACGCTTTGGTCTCT-3’, Rev 5’-CCCAGTTTGGAGAAGCCCAT-3’; *Slc1a2*: For 5’-ATCACTGCTCTGGGAACTGC-3’, Rev 5’-ACGAATCTGGTCACACGCTT; *Slc1a3*: For 5’-GAGAGATTGCAGCAAGGGGT-3’, Rev 5’-ATACGGTCGGAGGGCAAATC-3’.

### Immunohistochemistry

For IHC, transcardiac perfusion was carried out on 5 WT and 5 *Gnasxl*^m+/p-^ (KO) littermates (2-4 months old females), brain tissues were further fixed overnight in 4 % PFA/PBS and then dehydrated in 30 % sucrose/PBS. Postnatal brains were fixed without perfusion. 12 μm brain sections were prepared on a Leica CM 1950 cryostat. Sections were treated for antigen retrieval by submerging slides in 10 mM sodium citrate at 60 °C for 2 min and endogenous peroxidase activity was quenched by submerging section in methanol/0.3 % H_2_O_2_ for 5 min. Sections were blocked with PBS/10 % goat serum/0.25 % Triton-X100, incubated overnight with rabbit polyclonal anti-Gfap (Dako, Z0334, 1:3000) or mouse monoclonal anti-Nestin (BD Pharmingen, 556309, 1:100) antibody and developed using the VECTASTAIN Elite ABC Kit (Vector Laboratories) with 3,3’-diaminobenzidine substrate. Stained sections were dehydrated, permanently mounted in Eukitt (Fluka Analyticals) and imaged on a Leica microscope and LAS software (Leica Microsystems). Hypothalamic Gfap cell counts were obtained using ImageJ from images taken at 100 x magnification, whereby the 3^rd^ ventricle was positioned at the centre of the image. All areas along the dorso-ventral axis of the hypothalamic ventricle were included at equal left-right distance from the midline. Sections covered the rostro-caudal axis of the hypothalamus corresponding to the mouse brain atlas plates 36–50 (Bregma -0.58 mm to -2.30 mm) [60], including the PVH, ME, Arc, VMH, DMH and PH. Images from WT and KO littermates that matched to the same atlas plate were paired and processed for comparative glial cell counts. In total, 22 pairs of sections from the 5 WT/5 KO adult brains were included. For postnatal day 15, Gfap cell numbers were established from 33 pairs of sections from 3 WT/3 KO littermates from two litters. Images of adult hippocampus Gfap expression were taken from the same series of stained sections as the hypothalamus images, covering the mouse brain atlas plates 41–49 (Bregma -1.22 to -2.18 mm) [60]. In total, 15 pairs of sections from 5 WT/5 KO mice were analysed. For immunofluorescence, the same anti-GFAP and a chicken anti-Vimentin antibody (Millipore, AB5733, 1:4000) were used, combined with Alexa Fluor 488 donkey anti rabbit (Life Technologies) and DyLight 594 donkey anti-chicken (Jackson ImmunoResearch Laboratories) secondary antibodies (1:2000).

### Statistical analyses

Hypothalamic cell counts and qRT-PCR data were analysed by Student’s independent *t*-test. RNAseq statistical analysis was undertaken as described above. *P*-values in Additional file 1 are derived from the corresponding *q*-values following multiple hypotheses correction. All graphs show data as means ± SEM.

### Ethics approval

All animal (mouse) work was approved by the Animal Welfare and Ethical Review Body of the University of Liverpool and carried out in accordance with the revised UK Animals (Scientific Procedures) Act 1986 (UK Home Office Project Licence PPL40/3577) and the directive 2010/63/EU for Europe.

### Consent for publication

Not applicable.

### Availability of data and materials

The RNAseq data have been deposited in the ‘Gene Expression Omnibus (GEO)’ database under accession number GSE75878. Further datasets supporting the conclusions of this article are included within the article and its additional files.

## Additional files

Additional file 1: RNAseq list of gene expression changes in hypothalamus samples from *Gnasxl* ^m+/p-^ mice versus WT littermates. Genes are listed by their log2(fold change) from most up-regulated to most down-regulated. (XLSX 5507 kb) Additional file 2: Functional enrichment analysis using the DAVID bioinformatics resources. The whole mouse genome was used as background with default parameter settings. Full results, including *p*-values after correcting for multiple hypotheses testing, are shown. Statistically significant enriched categories amongst overexpressed genes related to the two broad categories of ‘ribosomes/rRNA binding and translation’ and ‘mitochondria/electron transport chain’. Amongst the down-regulated genes no statistically significant categories were found. (XLSX 166 kb) Additional file 3: Gfap expression in the hippocampus of adult *Gnasxl*-deficient mice is not changed. IHC for Gfap on coronal brain sections from WT (**A**, **C**, **E**) and KO (**B**, **D**, **F**) littermates at different rostro-caudal levels. In contrast to the hypothalamus, quantification of Gfap-expressing cells in the hippocampal areas did not show any significant difference (WT = 769 ± 16 vs KO = 746 ± 19 cells/section ± sem, *p* > 0.05, *t*-test, *n* = 15 matched pairs of sections from 5 WT and 5 KO mice). Scale bar = 100 μm. (PDF 731 kb) Additional file 4:
*Gnasxl* is not expressed in the ependymal layer, but the parenchyma of the hypothalamus. IHC (**A**) for XLα_s_ (green) in the postnatal day 1 (P1) hypothalamus and *in situ* hybridisation for *Gnasxl* at P4 (**B**) show scattered positive cells in the DMH, but not in the ependymal layer (ep) of the 3^rd^ ventricle (DAPI nuclear counterstain). (**C**) Similarly, IHC for XLα_s_ in the adult hypothalamus marks neurons in the parenchyma, but no expression is found in ependymal cells (arrow). XLα_s_ is a membrane-associated protein detectable in neurites, but no tanycyte extensions are stained. Arc = arcuate nucleus, DMH = dorsomedial nucleus, ME = median eminence. Scale bar = 100 μm. (PDF 619 kb) Additional file 5: Expression levels of glial solute transporter genes in hypothalami of adult *Gnasxl* ^m+/p-^ mice. qRT-PCR analyses did not detect any significant changes in expression levels of the glucose transporter *Slc2a1* (*Glut1*) or the excitatory amino acid transporters *Slc1a2* (*Glt1*) and *Slc1a3* (*Glast1*). (*N* = 6 WT and 6 KO samples; *t*-test, *p* > 0.05 n.s.). (PDF 273 kb)

## Abbreviations

Arc: arcuate nucleus
DMH: dorsomedial hypothalamic nucleus
Gα_s_: G-protein α-stimulatory subunit
GEO: gene expression omnibus
ME: median eminence
IHC: immunohistochemistry
KO: knock-out
NPY: neuropeptide Y
PH: posterior hypothalamic area
POMC: pro-opiomelanocortin
PVH: paraventricular hypothalamic nucleus
rRNA: ribosomal RNA
SCN: suprachiasmatic nucleus
SNS: sympathetic nervous sysem
VMH: ventromedial hypothalamic nucleus
WT: wild-type
XLα_s_: extra-large G-protein α-subunit
