# Supplementary figures and images for: Reductions in hypothalamic Gfap expression, glial cells and α-tanycytes in lean and hypermetabolic Gnasxl-deficient mice
Source: Mol Brain. 2016 Apr 14;9:39. doi: 10.1186/s13041-016-0219-1 (PMC4832494; doi:10.1186/s13041-016-0219-1)

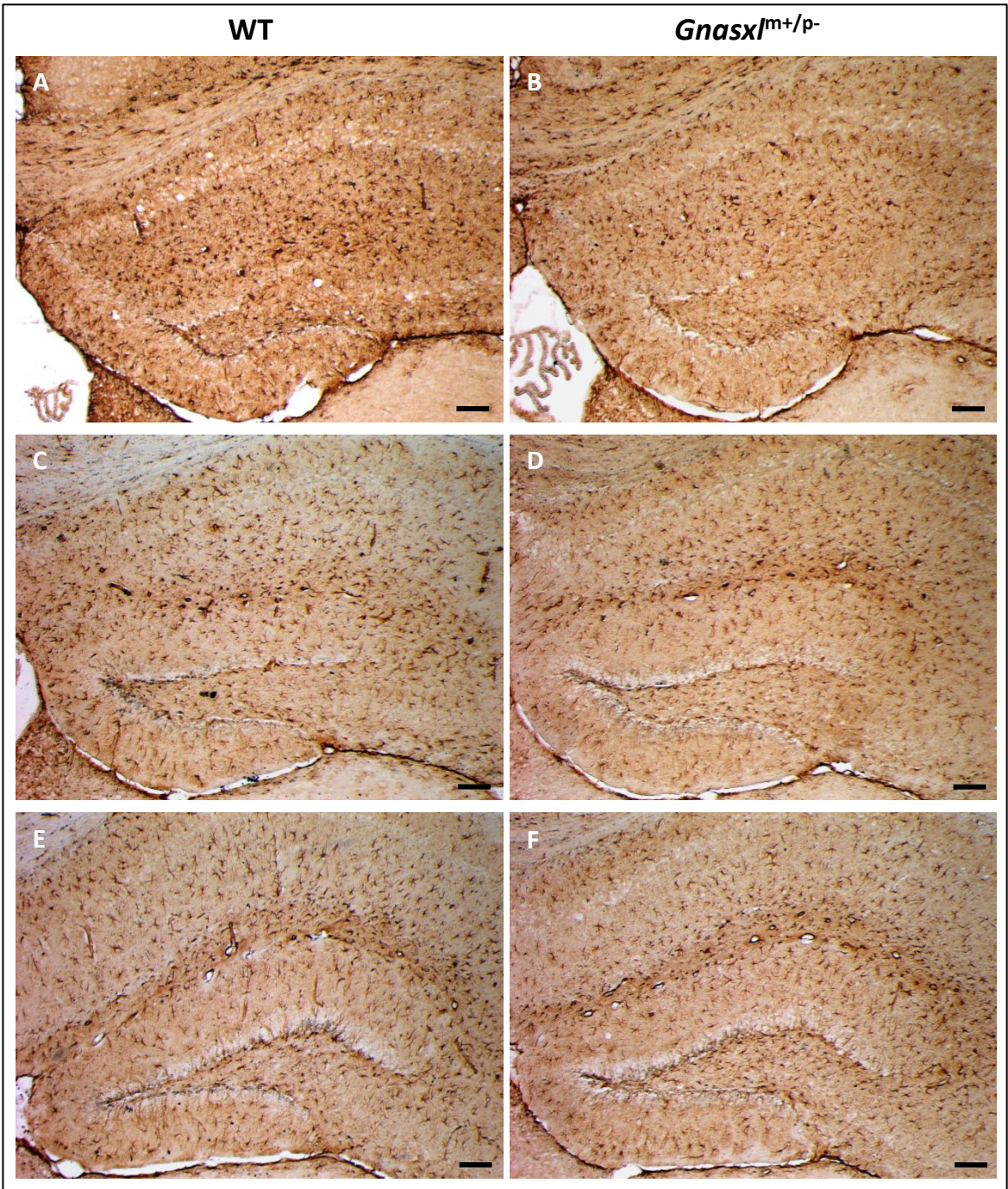

Additional File 3

Supplement: Additional file 3: — Gfap expression in the hippocampus of adult Gnasxl-deficient mice is not changed. IHC for Gfap on coronal brain sections from WT (A, C, E) and KO (B, D, F) littermates at different rostro-caudal levels. In contrast to the hypothalamus, quantification of Gfap-expressing cells in the hippocampal areas did not show any significant difference (WT = 769 ± 16 vs KO = 746 ± 19 cells/section ± sem, p > 0.05, t-test, n = 15 matched pairs of sections from 5 WT and 5 KO mice). Scale bar = 100 μm. (PDF 731 kb) [file 13041_2016_219_MOESM3_ESM.pdf]

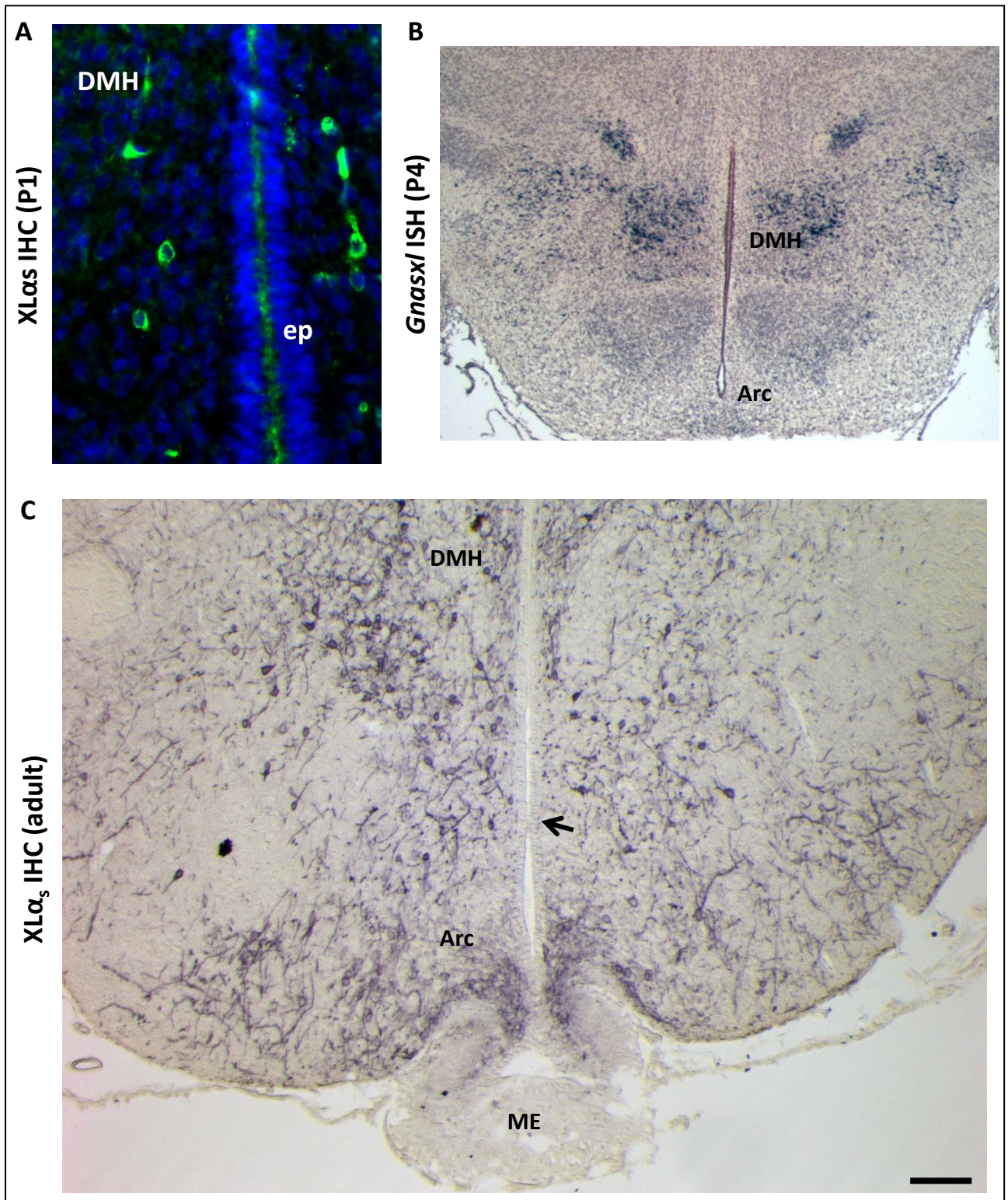

Supplement: Additional file 4: — Gnasxl is not expressed in the ependymal layer, but the parenchyma of the hypothalamus. IHC (A) for XLαs (green) in the postnatal day 1 (P1) hypothalamus and in situ hybridisation for Gnasxl at P4 (B) show scattered positive cells in the DMH, but not in the ependymal layer (ep) of the 3rd ventricle (DAPI nuclear counterstain). (C) Similarly, IHC for XLαs in the adult hypothalamus marks neurons in the parenchyma, but no expression is found in ependymal cells (arrow). XLαs is a membrane-associated protein detectable in neurites, but no tanycyte extensions are stained. Arc = arcuate nucleus, DMH = dorsomedial nucleus, ME = median eminence. Scale bar = 100 μm. (PDF 619 kb) [file 13041_2016_219_MOESM4_ESM.pdf]

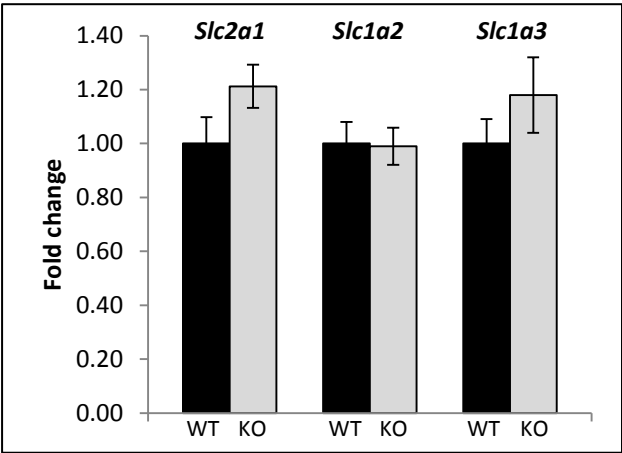

Additional File 5

Supplement: Additional file 5: — Expression levels of glial solute transporter genes in hypothalami of adult Gnasxl m+/p- mice. qRT-PCR analyses did not detect any significant changes in expression levels of the glucose transporter Slc2a1 (Glut1) or the excitatory amino acid transporters Slc1a2 (Glt1) and Slc1a3 (Glast1). (N = 6 WT and 6 KO samples; t-test, p > 0.05 n.s.). (PDF 273 kb) [file 13041_2016_219_MOESM5_ESM.pdf]
